# Protein–Protein Interactions in Food Systems: Analytical Advances and Quality Implications

**DOI:** 10.3390/foods15122072

**Published:** 2026-06-08

**Authors:** Muhammad Abdul Haseeb, Anna Wang, Ligen Wu, Muhammad Arif Ramzan, Mah E. laqa Taseer

**Affiliations:** 1 School of Food Science and Technology, Henan University of Technology, Zhengzhou 450001, China; mabdulhaseeb326@gmail.com (M.A.H.);; 2 National Engineering Research Center of Wheat and Corn Further Processing, Henan University of Technology, Zhengzhou 450001, China; 3 Institute of Chemical Sciences, Bahauddin Zakariya University, Multan 60800, Pakistan

**Keywords:** protein–protein interactions, food processing, protein structure, functional properties, customized nutrition, analytical techniques

## Abstract

Protein–protein interactions (PPIs) represent one of the major factors determining structure, function and quality in food products, especially in the case of industrial processing. Within complex food matrices, the structural and physical behavior of food components is controlled by PPIs that determine aggregation behavior, network formation, phase stability, and structural integrity and are thus directly related to the stability of the final product and how well a product may perform during a process. Recent developments in analytical techniques have facilitated the elucidation of PPIs and their application in activity-induced structural changes, in particular during thermal, non-thermal, enzymatic, and mechanical processes. In lieu of providing an exhaustive summary, this review synthesizes research evidence and findings related to measuring PPIs from main food systems, namely dairy, meat, cereal and plant-based products. The impact of different processing methods on PPIs and related quality characteristics including structure, stability and functional activity is critically assessed. Knowledge gaps and methodological limitations (in particular concerning laboratory scale industrial processes) are highlighted. By combining mechanistic considerations with practical performance considerations, this review allows us to rationalize the improvement of food processing strategies and to develop protein-based foods with better quality and performance stability.

## 1. Introduction

The 21st-century food system faces unprecedented challenges, among them feeding the 9.7 billion people projected for 2050, countering malnutrition for more than 2 billion individuals, and attaining sustainability milestones while preserving organoleptic quality. Proteins, which make up 10–20% of the majority of foods, are crucial for determining nutritional contribution, texture, stability, and acceptance by consumers. The functionality of food proteins arises mainly from protein–protein interactions (PPIs), both homotypic and heterotypic, that form hierarchical structures ranging from nanometer-sized aggregates to macroscopic networks.

Recent developments in analytical techniques, computational approaches and food processing technologies fundamentally changed food scientists’ capabilities for characterizing, predicting and controlling PPIs at the molecular and supramolecular level [1]. High-resolution analytical tools allow the real-time monitoring of the dynamic changes of proteins during processing while computational approaches aid in the rational design of components for proteins with well-defined functional properties [2].

Food proteins are macromolecules defined by their primary amino acid sequence, which determines their three-dimensional folded conformation and, consequently, their surface charge distribution, hydrophobicity, and capacity to form inter- and intramolecular bonds [3]. The principal physicochemical properties governing food protein behavior include solubility (pH- and ionic strength-dependent), surface hydrophobicity (directly correlated with emulsification and foaming capacity), denaturation temperature (a function of structural stability), and net charge at a given pH relative to the isoelectric point. Key techno-functional properties derived from these physicochemical characteristics include gelation (the formation of a three-dimensional protein network upon heating or acidification), emulsification (adsorption and stabilization of oil–water interfaces), foam formation and stability, water-holding capacity, and viscosity [4]. These properties are not intrinsic constants but are process-dependent: the same protein can produce fundamentally different structures and functional behaviors depending on pH, temperature, shear, and the presence of co-solutes. Understanding how processing conditions modulate these properties through changes in protein–protein interactions is therefore central to rational food product design [1].

## 2. Literature Search and Selection Criteria

This improved mechanistic understanding helps the development of management strategies to manipulate PPIs so as to improve nutritional composition, sensory attributes, shelf life or functional characteristics. Simultaneously, these innovations match the changing consumer preferences towards plant-based proteins, clean label formulations and sustainable alternatives—requiring a more in-depth understanding of how PPIs control the quality outcomes for different protein sources. This review presents a balanced view of the research published on PPIs and food quality between 2020 and 2026. We discuss: (1) cutting-edge analytical techniques for the description of PPIs within complex food systems; (2) protein aggregation, gelation, and network formation mechanisms at the molecular level; (3) impacts of traditional and new processing technologies on PPI behavior; (4) application across major food segments with focus on novel protein sources; (5) translation from fundamental science to industrial implementation and deployment; and (6) perspectives for the future involving computational design, sustainability, and personalized diet. By integrating recent literature, we aim to provide food scientists, nutritionists, and food technologists with a comprehensive understanding of the existing knowledge related to PPIs and food quality, and to identify fruitful avenues for further study.

The literature reviewed in this paper was identified through systematic searches of the Web of Science, Scopus, and PubMed databases, using the following search terms: “protein–protein interactions food systems,” “food protein aggregation,” “food protein gelation,” “dairy protein interactions,” “meat protein processing,” “plant protein functionality,” and “food processing protein structure.” The search was restricted to peer-reviewed articles, reviews, and book chapters published between January 2020 and March 2026. Articles were included if they directly addressed PPIs in food matrices, characterization methods, processing-induced structural changes, or quality implications. Studies conducted exclusively on pharmaceutical or medical protein systems without food relevance were excluded. A total of approximately 94 references meeting these criteria were retained for analysis.

## 3. Analytical Techniques for the Characterization of PPIs

Understanding protein–protein interactions (PPIs) in food systems requires a comprehensive, multiscale framework that integrates molecular mechanisms, macroscopic food quality characteristics, and consumer perceptions. Although a wealth of literature describes various aspects related to PPIs, from atomic-level bond formation to specific product applications, a systematic integration at the spatiotemporal scale is lacking. This section proposes a conceptual framework that clearly links molecular-scale phenomena to mesoscopic-scale structural assembly, functional properties, quality phenomena, and ultimately consumer acceptance. This framework will serve as an organizing principle for subsequent chapters and will provide a foundation to address the need to bridge the gap between mechanisms and industry in PPI research. The structural complexity of food matrices requires advanced analytical strategies to characterize PPIs at multiple length scales. Table 1 summarizes the analytical techniques used in PPI studies, their key applications, and interaction characterization capabilities. A range of diverse, complementary techniques has supported the latest research efforts, with the leading techniques being spectroscopic methods (34%), mass spectrometry-based proteomics (28%), high-resolution microscopy (22%), and modeling (16%) [5].

### 3.1. Hierarchical Organization of PPI Effects: Experience from Molecules to Consumer

**Figure 1:**
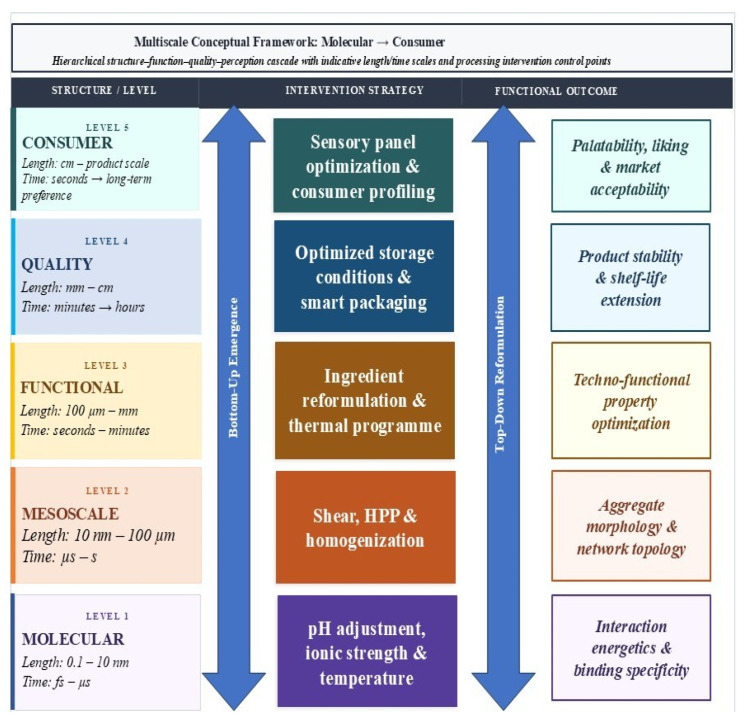
Multiscale conceptual framework linking protein–protein interaction (PPI) mechanisms to consumer-perceivable food quality attributes. The framework organizes PPIs across five hierarchical levels: atomic bonding (Level 1; 0.1–10 nm, fs–µs) through mesoscale assembly (Level 2), techno-functional network formation (Level 3), macroscopic quality attributes (Level 4), and organoleptic consumer perception (Level 5; cm–product scale). Bidirectional axis labels denote bottom-up emergence of structural organization from molecular interactions and top-down reformulation by which processing parameters engineer desired quality endpoints. G′ = storage modulus; WHC = water-holding capacity; HPP = high-pressure processing; DF = fractal dimension; EAI = emulsifying activity index; TBARS = thiobarbituric acid reactive substances.

The effects of protein–protein interaction on food systems can be traced through five independent yet interconnected levels, each with its own spatial and temporal scales and mechanisms of action (Figure 1). This hierarchical structure provides a rigorous model for analyzing how atomic-scale interactions work through a series of structural combinations to ultimately influence consumer perception and acceptance.

### 3.2. Molecular Scale (Atomic-Level Interactions)

At the molecular level, with molecular lengths ranging from 0.1 to 10 nanometers and timescales from picoseconds to microseconds protein–protein interactions (PPIs) are regulated by a series of fundamental intermolecular forces, including hydrogen bonds, electrostatic interactions, hydrophobic interactions, dissociation interactions, and covalent disulfide bridges. The free energy distribution revealed at this resolution controls the thermodynamic stability of the associated proteins and the accessibility of the kinetic pathways for their formation. Furthermore, environmental factors such as pH, ionic strength, temperature, and water activity affect the relative balance of these forces on proteins by influencing surface charge distribution, hydration layer remodeling, and conformational flexibility.

At the molecular level, interactions include both reversible intermolecular interactions without covalent bonds and irreversible changes in intermolecular interactions through covalent bonds. Non-covalent protein–protein interactions are regulated by dynamic equilibrium, characterized by binding rate constants (k_on) and dissociation rate constants (k_off), with the equilibrium dissociation constant (Kd) ranging in value from nanomolar to millimolar scales, depending on the strength of the interaction [3]. Coordination changes, particularly disulfide bonds formed during catalysis or thermal treatment of protein disulfide isomerases, lead to permanent cross-linking with bond energies of approximately 250 kJ/mol, significantly altering the overall interaction pattern of proteins. Molecular dynamics simulations conducted between 2023 and 2025 highlighted the crucial role of transient collisions (lasting several nanoseconds) in the conformational selection mechanisms of long-term assembly pathways. These results suggest that kinetic intermediates should be considered in analyses, rather than focusing solely on equilibrium structures.

#### 3.2.1. Mesoscale Structure (Aggregate and Network Formation)

Protein–protein interactions (PPIs) at the molecular level can spontaneously assemble into mesoscale structures ranging in length from 10 nanometers to 10 micrometers and in temporal scales from milliseconds to hours. These structures include dimers, oligomers, fibrils, aggregates, and three-dimensional structures. The existence of this hierarchical structure is due to the spatial and temporal coordination of individual protein-binding events. The network topology depends on a delicate balance between attractive and repulsive forces, the symmetry of binding sites, and the inherent flexibility of protein molecules [14].

The transformation of monomeric proteins into macroscopic networks depends on processing conditions and protein concentration. Below the critical gel concentration (CGC), proteins form soluble aggregates through limited affinity. Once the CGC is exceeded, the transformation of these isolated aggregates into a network spanning the entire system can be well described by percolation theory, where the gel threshold depends on molecular weight, surface charge, and protein disorder. The fractal dimension (DF) associated with protein aggregates controls their network density and mechanical properties, with values ranging between approximately 1.8 for diffusion-limited clusters and approximately 2.1 for reaction-limited clusters. Recent studies using small-angle X-ray scattering (SAXS) and ultrasmall-angle neutron scattering (USANS) have clearly demonstrated that processing-induced changes in DF are highly correlated with macroscopic structural properties, thus establishing a quantitative relationship between structural properties and function [5].

Importantly, mesoscale structure is not a passive consequence of molecular interactions, but rather actively regulates subsequent protein–protein interaction (PPI) formation through mechanisms such as crowding effects, confinement phenomena, and exclusionary volume interactions. In crowded protein networks, macromolecular crowding can reduce the effective diffusion coefficient of proteins by up to two orders of magnitude, and the association equilibrium shifts towards complex formation due to the contribution of entropy. This interdependence between mesoscale organization and molecular interactions leads to path-dependent assembly processes, where processing history determines the final network structure.

#### 3.2.2. Functional Properties (Techno-Functional Attributes)

Mesoscale protein networks can be translated into certain functional properties (length scale: 10 micrometers to 1 mm; time scale: seconds to days), which determine food processing behavior and product performance. These technical properties, such as water retention, emulsification, foaming, gelling, and texture formation, are not the result of individual intermolecular interactions, but rather the result of the combined effects of the overall mechanical and interfacial properties of the protein network.

Water-holding capacity in protein networks exemplifies the structure–function relationship. Water retention depends on capillary pressure within the corresponding interstitial pores (following the Young–Laplace equation), osmotic pressure generated by the ion-loaded protein surface (characterized by the Donnan equilibrium equation), and entropy loss experienced by water molecules within the confined geometry [1]. The size distribution of network pores (directly controlled by the mesoscopic structural arrangement) dominates water movement within the network: pores smaller than approximately 100 nm effectively retain water molecules, while pores larger than approximately 1 mm allow water molecules to easily escape under gravity or mechanical forces. Quantitatively, water-holding capacity decreases with increasing average pore size and network surface charge density, the latter estimated using mesoscopic descriptor parameters obtained by confocal laser scanning microscopy (CLSM) or atomic force microscopy (AFM) [15].

Emulsification and foaming are key interfacial functional properties, in which protein–protein interactions (PPIs) play a multifaceted role: firstly, regulating the kinetics of protein adsorption; secondly, controlling conformational rearrangement at the interface; and thirdly, controlling the structure of the interfacial network. The cohesive stability of protein-stabilized emulsions depends on the mechanical strength of the interfacial protein film, which in turn reflects the degree of PPI-mediated crosslinking at the oil–water or gas–water interface. Recent studies using interfacial shear rheology have shown that disulfide bonds formed at these interfaces can increase the interfacial elastic modulus from approximately 1.0 to 5.0, thereby improving emulsion stability and enhancing the foam’s resistance to drainage and erosion [16].

#### 3.2.3. Quality Attributes (Product Performance)

The accumulation of the functional properties is responsible for the quality parameters that can be observed, with a length scale ranging from 1 mm to 10 cm and a temporal domain ranging from hours to months, such as texture, appearance and flavor release, nutritional bioavailability, and shelf stability [17]. These product-level attributes are the result of the synergistic combination of many of these functional properties and their interactions with the non-protein constituents (lipids, carbohydrates, minerals, phenolics). Importantly, linkages between functional properties and quality metrics are often non-linear and context-dependent; small perturbations in network structure may engender disproportionate changes in quality outcomes.

Texture perception, usually considered one of the most important attributes of quality, is a property resulting from mechanical response of protein networks under the forces exerted during mouth processing. The stress–strain curve displayed by protein gels during mastication directly determines sensory parameters such as firmness, cohesiveness and springiness that are assessed quantitatively through texture profile analysis (TPA). Nevertheless, the interactions between network rheology and sensory perception are complex: e.g., a rise in elastic modulus of the gel can lead to an increase in firmness, but simultaneous decrease in perceived juiciness due to a possible decrease in water-holding capacity (WHC) associated with a densification of the network. Consequently, this interdependence of quality attributes requires the use of multi-objective optimization strategies for the design of PPI networks for particular product targets.

In the context of food engineering, the kinetics of flavor release is an important quality attribute that is strongly controlled by protein–protein interaction (PPI) mediated network architecture. The partitioning behavior of aroma constituents between the proteinaceous matrix and the aqueous or gaseous phase, which is contiguous to the matrix, is determined by inherent hydrophobicity and specific affinity for definable binding sites in the protein matrix. Highly cross-linked dense networks create retarded diffusion of the volatile entities, thus retarding flavor release during consumption, and can contribute to heterogeneous flavor perception. Conversely, networks with larger pore sizes and weaker PPI interactions facilitate rapid aroma release but may compromise stability during storage. Recently, quantitative structure–activity relationships (QSPRs) using machine learning methodologies have become powerful predictive tools providing a tool for converting the mesoscale structural descriptor into anticipated, predicted trajectories of the flavor release not only to support rational engineering of protein networks with desired sensory attributes [18].

#### 3.2.4. Consumer Perception and Acceptance

Ultimately, quality attributes are appraised by the episodes of the consumer sensory perception and acceptance (spatial extent: entire product; temporal extent: period of consumption episode till the establishment of long-term preference). At the top of this hierarchy of evaluation, there is a synthesis of objective quality measures with the subjective values of human responses, cultural norms and individual preferences. Consumer acceptance is not only based on intrinsic factors of the product per se, but also on extrinsic factors including visual appeal, packaging design, branding strategy or health-related claims. Nonetheless, textural and taste characteristics of foods resulting from protein–protein interaction (PPI) remain as the main drivers of repeat purchase behavior as well as long-term market viability.

The link between protein–protein interaction (PPI) modification and consumer acceptance is particularly strong with plant-based protein products where differences in amino acid composition and structural properties of plant-based proteins from animal ones give rise to unique sensory characteristics. Plant proteins typically produce matrices with altered mechanical properties and release kinetics of flavors which, in animal protein textures, are often recognized by consumers as beany, grainy or chalky flavor. Recent studies on consumer acceptance have revealed that the acceptance of plant-based meat alternatives increases significantly if the textural properties (directly controlled by the PPI engineering through processing methods such as high-emulsification at high moisture content extrusion) resemble those of the conventional meat products. Quantitatively speaking, consumer preference scores are associated with certain texture attributes (hardness: 20–40 N, springiness: 0.75–0.85, chewiness: 15–30 N-mm) which can be achieved through optimized PPI manipulation [19].

Nutritional bioavailability is another aspect of consumer perception which is tightly connected to the protein–protein interaction (PPI) mediated network architecture. The digestibility of proteins and the bio accessibility of constituent amino acids depend on the susceptibility of these networks to proteolytic enzymes, which in turn is determined by such parameters as the density of the network, typology of cross-linking, and the accessibility of the enzymatic cleavage sites in spatial terms [20]. In cases of excessive cross-linking, whether by formation of disulfide bridges or by enzyme-mediated modification, one finds a marked reduction in in vitro protein digestibility, that in some cases is of the order of 90% or more, and in other cases is less than 60%. Such a reduction can compromise the nutritional value of the product, whilst textural attributes are ostensibly enhanced [21]. Consequently, apparent enemies between desirable textural characteristics and optimal nutritional outcomes highlight a need to develop multi-scale optimization strategies which include consumer priorities covering a scale of quality dimensions.

### 3.3. Integrative Principles: Scale-Bridging Mechanisms

The hierarchical framework provides a vertical organization on the scales; however, in order to understand the effects of protein–protein interaction (PPI) in food systems, it is imperative to explicitly consider the mechanisms that act as bridges between neighboring scales. These scale-bridging mechanisms work in both bottom-up (emergence) and top-down (constraint) directions and, therefore, set up a complex network of feedbacks rather than a typical linear cascade of events.

#### 3.3.1. Molecular Interactions to Macroscopic Properties

The emergence of collective behavior at the macroscopic level is fundamentally the result of individual binding events at the molecular scale. In PPI systems this is demonstrated through the evolution of molecular binding events to the mechanical features of the obtained network, which are not derivable from the single-molecule characteristics. For instance, the gel elastic modulus of a protein network, which is a mesoscale property, depends not only on individual protein–protein bond strength but also on network topology, cross-link density, and strand flexibility. Consequently, computational models that combine molecular dynamics and continuum mechanics are needed to capture these dependencies.

A rigorous quantitative account of bottom-up emergence may be stated using coarse-graining techniques but a formal account of such coarse-graining requires a systematic averaging over quick molecular degrees of freedom, thereby giving rise to effective interactions operating at larger and larger scales. For example, high-resolution atomistic simulations of molecular dynamics of protein/protein associations could be reduced to emergent effective potentials that can be represented by mesoscale (Brownian dynamics) aggregate formation simulations, which in turn may be further reduced to finite element formulations of macroscopic deformation of hydrogel matrices [22]. Such multi-scale computational paradigms, which are increasingly supported by experimental data sets from synchrotron X-ray scattering and neutron spectroscopy, provide the power to anticipate processing outcomes from fundamental, protein structural biophysical information.

#### 3.3.2. Macroscopic Conditions Modulating Molecular Behavior

In protein-dense matrices, macroscopic variables (e.g., network elastic modulus, pore size distribution) impose spatial constraints on protein diffusion and conformational dynamics, fundamentally altering PPI kinetics compared to dilute solution behavior. Similarly, macroscopic control parameters can be found in processing variables (such as temperature, pressure, and shear rate) in the molecular energy landscape, which can alter equilibrium positions and affect reaction pathways.

An example of top-down constraints is macromolecular crowding. In practical food systems protein concentration is 50–200 g·L^−1^, which is far in excess of dilute regimes usually studied in laboratory biochemistry. Such elevated levels create some excluded volume effects that stabilize compact protein conformations and favor association reactions [23]. Indeed, the fact that the effective concentration that a protein is exposed to locally within the crowded network can be 10–100 times greater than the bulk average concentration, drastically increases the rate at which PPIs are formed and shifts the control from kinetics to thermodynamics. In constrained geometries (such as the interfacial layers of emulsion droplets or foam sheets), crowding is further amplified, where interfacial curvature and proximity effects impose additional restrictions on protein behavior.

### 3.4. Critical Control Points for Targeted PPI Manipulation

Hierarchical framework and scale-bridging mechanisms (Figure 1) reveal different control points in which targeted interventions can be used to closely control PPI behavior to achieve desired food quality outcomes. These critical control points operate at different scales and have varying degrees of precision and reversibility.

#### 3.4.1. Protein Engineering and Chemical Modification

Protein sequence and structure is the most fundamental locus of control that can be accessed by genetic engineering, enzymatic refinement or chemical derivatization. Rational design of protein variants, through changes in patterns of surface charge, hydrophobicity or cysteine content, allows for the specific tuning of the intermolecular interactions. For example, the introduction of negatively charged residues into the surfaces of plant proteins increases the electrostatic repulsive force, which will improve the undesirable aggregation during thermal processing while maintaining the pH-dependent gelation capacity [24]. Site-ordered and computational design algorithms with site-directed mutagenesis make it possible to predict and optimize the binding affinities of proteins to proteins almost to the level of atoms.

At the molecular level, surface modifications such as phosphorylation, glycosylation and acylation fundamentally change the physicochemical surface characteristics of the proteins, hence engendering novel forms of interaction. For instance, casein proteins when phosphorylated increase their calcium binding affinity while at the same time adjusting the stability of the casein micelles, resulting in the significant impact on the textural characteristics and overall stability of dairy products. Enzymatically catalyzed cross-linking organized by transglutaminase leads to isopeptide formations between glutamine and lysine residues that forge persistent polymeric networks that are considered to be more resilient from mechanical perspectives and are resistant to excessive heat [25]. The fine precision of these molecular interventions allows the targeted improvements of certain functional properties coupled with the reduction of negative consequences on other parameters of ancillary quality.

#### 3.4.2. Environmental Modulation of PPI Pathways

Temperature, pH, ionic strength, and mechanical forces are the main and easy-to-manipulate parameters that affect protein–protein interactions by their modulatory influences on protein conformation, charge distribution, and solution thermodynamics. Notably, the manipulation of temperature has a multifaceted effect and it simultaneously modulates the kinetic process of protein unfolding, the rates of covalent bond formation, as well as the structural organization of aggregates, forming a versatile tool for the selection of the myriad potential routes of assembly. In the case of whey protein solutions, such heating at 70 °C causes a controlled limited unfolding which allows disulfide exchange and so fuels the formation of fine striated network structures reminiscent of the native assembly. Conversely, if exposure is made at 90 °C, extensive unfolding occurs in which dense particulate aggregates with substantially different textural features are produced.

In the context of protein chemistry, the modulation of pH provides a sophisticated way of exerting electrostatic governance over protein–protein interactions (PPIs) through the process of modifying the net surface charge of the subject macromolecules. At or in the immediate vicinity of the isoelectric point (pI), proteins have an almost nil electrostatic repulsion, allowing for an increased tendency of protein aggregation, which then has a tendency to form large, density-rich networks with significant elastic moduli. In contrast, the solution pH values deviated from the pI, which causes the powerful electrostatic repulsion, effectively stabilized the proteins against unwanted aggregation and may reduce the gelation efficiency. The strong pH-dependence of protein–protein interaction provides a reversible control of the aggregation phenomena. Proteins are kept dispersed in solutions of pH far away from their isoelectric point (pI), but when the pH varies close to the critical value, the proteins quickly form viscoelastic gels due to the reduction in electrostatic repulsions [26]. Such a pH changeable mechanism is routinely exploited both in acid-induced dairy gel systems (e.g., production of yoghurt and cheese) and in heat-acid coprecipitation protocols (for protein recovery).

#### 3.4.3. Spatial Organization and Interface Design

Mesoscale architectural design represents an emerging power of control, where spatial organization of protein networks is intended for the sake of their conferring specific functional properties. High moisture extrusion and electrospinning allow protein networks to be aligned into fibrous structures resembling muscle tissue architecture and, therefore, to possess anisotropic mechanical properties that are characteristic of meat [27]. Layer-by-layer assembly and 3D printing makes it possible to fabricate structures with multi-scale hierarchical shapes with engineered mechanical gradients and compartmentalized functionalities [28]. These strategies go beyond the conventional bulk processing method in providing precise positional regulation of distribution and orientation of PPIs.

Interface engineering which allows for precise modification of protein assembly at oil–water, gas–water, or solid–liquid interfaces, and provides broader structural control. The conformational landscapes and interaction ways of proteins at these interfacial milieus are quite different from those of their bulk counterparts, due to orientational restriction and reduction of dimensionality and special interfacial forces. By systematically varying the interfacial protein concentration, adsorption kinetics, and the cross-linking chemistry, one can independently vary the mechanical properties of the interface, and stabilize emulsions and foams, besides regulating release profiles. Recent advances in interfacial shear rheology and sum frequency generation spectroscopy are now presenting a new opportunity for quantitative understanding of the architecture of interfacial protein networks, and therefore for the rational design of emulsion- and foam-based systems with predetermined performance properties [29].

### 3.5. Framework Application and Organizational Logic

Provided herein represents a comprehensive multi-scale conceptual structure that is the organizational backbone for the rest of this review. At the molecular scale, we focus on protein–protein interactions (PPIs), highlighting recent progress in trying to understand aspects of interaction energetics, kinetics and modulation by environmental parameters. Subsequently, we investigate processing technologies as tools to modulate PPIs on a molecular as well as a mesoscale level; this entails a detailed analysis of the translation of processing parameters into respective structural consequences. Finally, we ground the framework using specific food products, thereby showing how the functional properties, quality attributes, and consumer acceptance of food products vary based upon the kinds of changes that occur at the molecular and mesoscale level for a wide range of food products.

Crucially, this framework allows critical knowledge gaps which are not yet resolved despite the profusion of research at individual scales to be considered. Such gaps include a limited understanding of PPI kinetics in crowded environments, the lack of elaborate characterization of the role of processing history in the evolution of the networks, and the lack of robust predictive models to relate the mesoscale structure to sensory perception. These lack of knowledge boundaries define priority areas for future research. By explicitly using the multi-scaled framework to map the state of knowledge at present, in this review we aim to outline the gradual direction of research into integrated methodologies to achieve a seamless scaling from the foundation to the tangible applications of the modern food systems.

### 3.6. Comparative Scalability and Industry Translation

Although the methods discussed above allow for general analytical capabilities, their industrial applications differ widely. Conventional analytical techniques like surface plasmon resonance (SPR), isothermal titration calorimetry (ITC) and mass spectrometry offer excellent details on mechanisms yet are largely restricted to analysis due to the prohibitive cost of instrumentation, technical expertise required and low throughput [11].

In contrast, techniques based on the dynamic light scattering (DLS), differential scanning calorimetry (DSC) and size exclusion chromatography (SEC) have become successfully applicable in quality-control applications in the dairy, meat and plant-protein industries, because of their automation abilities and fast analysis time [30]. Fluorescence spectroscopy and circular dichroism (CD) being the oldest analytical tools, quickly showed process improvements through elucidation of structure–activity relationships and the selection of commercial parameters [7].

For general protein–protein interaction measurements, the best way to do this is to combine high-throughput analytical techniques (for instance, turbidity measurements, DLS) with additional methods that create mechanical validation. Future development should place emphasis on miniaturization, automation and combination of several detection modalities in order to address the gaps between laboratory estimates and production floor determination.

## 4. Molecular Mechanisms of PPIs in the Food System

**Figure 2:**
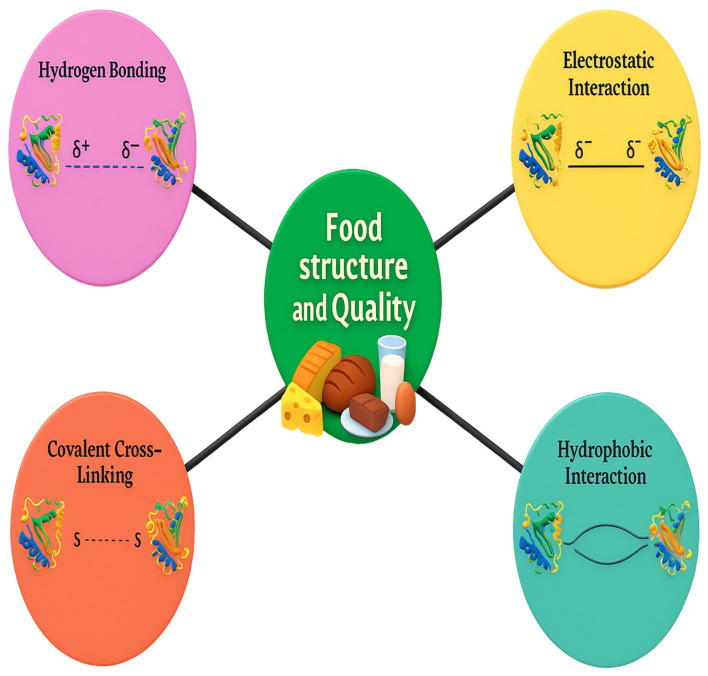
Molecular mechanisms of protein–protein interactions.

Knowledge of molecular mechanisms leads to the ability to exert rational control over food properties. New work is revealing novel information on forces emerging under PPIs and the organization of food systems hierarchically as described in Figure 2, which help to understand the subject more thoroughly.

### 4.1. Non-Covalent Interaction Mechanisms

Non-covalent interactions play major roles in protein functionality and food quality. Forces between hydrophobic residues are known to be one of the factors responsible for protein aggregation in heat-treated dairy foods and recent research has revealed how temperature can translate into conformational modifications to expose buried hydrophobic residues [31]. The advanced computational techniques have also been applied to the study of the hydrogen bond networks as they may play an important role in protein aggregation within gluten networks and are responsive to various processing conditions. Interplay among the diverse types of non-covalent interactions forms a major modulating factor of protein functionality in complex food systems by acting in a context-dependent manner in relation to the surrounding food matrices as well as processing conditions.

### 4.2. Covalent Cross-Linking in Food Matrices

Covalent cross-linking is an important mechanism for PPIs that irreversibly changes protein networks within foods. Enzymatic cross-linking, specifically through the use of transglutaminase, has been well investigated for the enhancement of textural properties of meat, dairy, and plant foods. It has been the focus of research to further optimize enzyme activity conditions and the impact of cross-linking patterns on resulting food attributes. Non-enzymatic cross-linking, encompassing Maillard-induced cross-links and disulfide bond formation, has been gaining prominence owing to its relevance to both thermal processing and protein-rich food stability under storage [32]. Novel analytical methodologies have allowed for detailed characterization of cross-links and impact on digestibility and nutritional quality. Optimizing the balance between enzymatic and non-enzymatic cross-linking has been found crucial for the provision of desirable food quality attributes.

### 4.3. Aggregation Kinetics and Network Formation

The aggregation kinetics of PPIs plays a major role in determining food structure formation and stability. Time-resolved methods have been used to follow aggregation kinetics in real time, demonstrating the presence of different phases during heat-mediated aggregation of milk proteins and environmental condition dependencies. The protein aggregation model based on the concept of nucleation-growth has been developed for different food proteins, paying particular scrutiny to factors affecting the critical nucleation step. Two-stage nucleation mechanisms involving primary amorphous precursor formation followed by structural rearrangement have been detected for a number of food protein systems. Competition and synergism among different protein types have been explored for protein systems containing two or more protein types, representative of real food matrices. These investigations have shown how protein composition impacts the aggregation pathways and final product properties [33]. Network formation and dissolution dynamics have important consequences for texture formation, stability during storage, and release of flavor compounds on eating. Advanced multi-type interaction and interaction kinetics-containing molecular models have facilitated greater predictability and control over protein behavior in complex food systems.

Despite significant advances, several unresolved issues remain. The relative contribution of non-covalent versus covalent mechanisms to network stability in real food matrices remains contested: some studies report dominant hydrophobic effects under moderate thermal stress, while others identify disulfide crosslinking as the rate-limiting step, with the balance appearing to be matrix- and protein-concentration-dependent. Molecular dynamics simulations, while increasingly informative, are typically conducted on isolated protein pairs in dilute aqueous environments, conditions that poorly represent the crowded, multi-component food matrix. Extending these models to realistic concentration regimes remains computationally prohibitive with current methods.

## 5. Processing Conditions’ Influence on PPIs and Food Quality

Processing conditions significantly impact PPIs and food quality characteristics, and each technology has different advantages and limitations for protein functionality manipulation. There are pros and cons for each technology for interacting with protein functionality, summarized in Table 2. The table highlights the latest analytical techniques used to characterize the dynamics and structural changes of protein–protein interactions (PPIs) and diagrammatically illustrates the various processing methods that modify PPIs, thereby influencing food quality characteristics such as texture, stability, and nutritive value..

### 5.1. Thermal Processing Effects

Heat processing remains high on the research agenda, and more recent work has probed the effects of varying heating regimes (rapidity vs. gradual heating) on protein denaturation and consequent aggregation behavior of dairy products [39]. Heat-induced protein complexes between whey proteins and caseins have been reported to greatly impact the textural and sensorial qualities of dairy products. Recent investigations have examined how controlled thermal gradients can drive selective protein complex assembly during heating, enabling the formation of protein structures with defined functional characteristics. Sequential denaturation of whey proteins under incremental temperature ramps (55 °C to 85 °C) has been shown to produce distinct aggregate populations with differing gel-forming capacities [40]. These findings suggest that precise temperature programming, rather than endpoint temperature alone, is the critical variable in determining both aggregate morphology and the resulting textural properties of the final product. Moreover, temperature gradients indeed have the potential to unclasp selective protein strands and thus can be utilized to construct protein complexes with designated capabilities.

So far as deriving these conclusions, several advanced spectroscopic methods have been implemented, which delve into the nature of heat and its role in the aggregation of proteins. The steps for the formation of disulfide bonds can be rearranged as first intramolecular adjustment and then intermolecular gap-bridging, which sets the strength of the stubborn gel [41]. The aggregation begins first with the soluble gel proteins while the gel network is initially forming. Calcium ions play a critical role in heat-induced protein reactions by exposing hidden binding sites, which facilitate the formation of protein interconnections.

For meat products, interactions between cooking temperature, protein unfolding, and water-holding capacity have been further detailed, emphasizing the contribution of myofibrillar protein interactions to tenderness and juiciness. Raising the temperature to 65 °C denatures tropomyosin/troponin, actinin denatures at 50 °C, and 58 °C denatures myosin in the head region, and all of these processes influence the texture of meat [42]. Heating techniques can help the protein unfold properly to keep more water in the meat and make it tender as desired. Cooking causes changes in both collagen and myofibrillar protein and research revealed that the best conditions ensure these two changes do not clash [43].

Similar underlying principles have been used to explain texture development within plant-based meat alternatives, for which controlled thermal unfolding and aggregation are crucial for attaining textural properties akin to the real thing. Time-temperature relationships impact significantly the nature and quantity of protein aggregation, as well as help to determine optimal processing conditions for product classes.

### 5.2. Microwave and Infrared Heating

Protein interactions have been studied with regards to their response to modern heating methods. Heating proteins in a microwave tends to cause different types of agglomeration since it preferentially heats the more polar areas of the protein molecule. Infrared heating resulted in some surface changes for proteins that formed self-assembled networks with enhanced properties. Protein aggregation may be controlled by observing the change in a protein’s electrical conductivity during its denaturation [40].

Similar to animal-derived proteins, plant-based proteins undergo certain texturizing processes pertaining to folding and binding that determine their texture. Plant proteins have a different sequence of amino acids and require longer, more extreme heating for aggregation compared to animal proteins [44].

In regard to controlling the amount of protein aggregates formed and the ideal processing parameters for different product classes, the relationship between time and temperature is critical. Kinetic modelling has yielded equations that explain the relationship between protein concentration, heating time, and temperature in relation to the results of gels formed [45]. Advanced protein research has shown that there are different activation energies for protein aggregation which suggests that proteins need to be heated according to their type. With real time monitoring of protein changes through fluorescence, the temperature control associated with food processing becomes easier to achieve the desired level of protein interactions [46].

### 5.3. High-Pressure Processing (HPP)

HPP is an innovative, non-thermal approach to food preservation with significant implications for PPIs. Novel investigations have reported protein unfolding and association mechanisms, under pressure, different from heat treatment. These investigations have demonstrated how pressure-induced PPIs can be utilized for the development of novel textures and improvements in functional properties that cannot be achieved through treatment with heat. The studies demonstrated how pressure above 400 MPa affects molecular bonds, stating that pressure does not denature proteins the same way heat does [47]. Pressure can unfold certain regions of a protein while keeping other regions tightly packed, facilitating the formation of new types of proteins.

Whey protein isolate gels created by the pressure method (600 MPa for 10 min) were more ductile and water-absorbent than those formed by heat treatment [48]. The behavior of plant proteins under pressure differs, identifying 500 MPa [49] as the ideal pressure for gelation of pea protein, whereas soy protein required 700 MPa. There is evidence suggesting that some conformational changes induced by pressure are not fully reverted upon pressure release, while others are [50].

Comprehensive studies demonstrated that simultaneous application of 400 MPa pressure and 60 °C temperature creates synergistic effects on protein unfolding, reducing processing time by 70% while improving final product quality [47]. Pressure-assisted thermal processing can achieve similar protein modifications to conventional thermal processing at significantly lower temperatures, preserving heat-sensitive nutrients and flavors.

HPP usage for modifying proteins in the manufacturing industry has grown in popularity. HPP processing was found to scale successfully for dairy alternative production. Commercial food processing research demonstrated the benefits of applying HPP to plant protein ingredients before incorporation into food, showing improved quality and reduced processing in production problems [51].

### 5.4. Enzymatic Modification

Enzymatic modification has gained interest for the purposeful modification of PPIs for desirable food characteristics. Transglutaminase cross-linking has been well studied for textural and stability improvement properties in a wide range of high protein foods, and its research has primarily focused on the improvement of enzyme activity in complex food systems [52]. Transglutaminases can now be used at higher food processing temperatures because they are more stable and compatible with a greater range of foods. Mutagenesis at one site on transglutaminase creates an enzyme which is more optimal at specific joining within proteins. Transglutaminase has seen little innovation, but other enzymes have been developed. According to the latest studies of protein, laccase enzymes create protein cross-links, which also provide antioxidant properties and enhance the color stability and sensory appeal of the resulting material. One study discovered that employing transglutaminase and protease, in a stepwise method, enabled the formation of protein networks with diverse properties. The use of encapsulated enzymes in food processing allows for their controlled release and activation at specific stages, thus controlling the modification of proteins during the process [34,53].

Proteolytic enzymes have been employed for site-directed peptide modification in which the peptides were modified for interaction properties. Even more, this technique has been applied to reduce allergenicity, improve digestibility, and augment other functional properties as emulsification and foam formation [54]. Recent investigations indicate that enzyme combinations can selectively reduce sensitizing protein epitopes while preserving technical functional properties [55]. This method causes low allergenicity and does not change protein architecture. Research indicates that controlled proteolysis can enhance the health-promoting functions of food proteins [56].

### 5.5. Emerging Non-Thermal Technologies (Ultrasound, Plasma, PEFs)

Emerging methods such as ultrasound, cold plasma, and PEFs (pulsed electric fields) are gaining attention due to their ability to modify PPIs without the application of harmful heat like traditional methods. Applying a pulsed electric field to cells changes the shape of proteins and therefore some parts of a protein are made accessible for interactions, thus altering the strength with which proteins can adhere to one another. Plasma at low temperature changes the amino acid side chains of some food proteins, which alters the structure of food protein assemblies, which in turn may affect how aggregation occurs as well as the capabilities of the proteins [57]. In emulsions, foams and gels, ultrasound can modify cavitation effects that assist proteins in unfolding, splitting to form smaller parts, or solidifying to form a single entity. Thus, with the application of these technologies, in addition to improving the color and texture of foods, there is less energy used, making it more sustainable. These technologies can induce conformational changes and alter interaction patterns that are different than what have been reported using traditional processing technologies [58]. Ohmic heating has also shown to produce new protein aggregation patterns due to the fact that it is a volumetric heating method as well as the probable electrochemical effects associated with the heating technique. Recently, protein networks formed under ohmic heating and their resulting influence on quality attributes were described [59]. The use of more than one processing approach, either applied successively or in combination, has identified to be effective in inducing desired characteristics in terms of PPI modification and properties of the final food. Further elucidation of synergism and antagonism among the combination treatments represents a continuing area of research with potential implications in the design and optimization of food products.

### 5.6. Implications for Processing Optimization

#### 5.6.1. Temperature–Time Window Definition Through PPI Dynamics

Protein–protein interaction kinetics directly reflect the formation of critical processing parameters. In thermal processing, knowing the starting temperature of aggregation and the denaturation rate allows the definition of precisely timed temperature compounds that achieve microbial safety while maintaining functional properties. For example, whey protein isolate (WPI) gelation requires temperatures above 70 °C to unfold β-lactoglobulin and initiate subsequent cross-linking, whereas temperatures above 90 °C for >5 min lead to irreversible gel aggregation and syneresis. By mapping these interfaces, producers can optimize the HTST conditions to 72 °C for 15 s, which is sufficient for viral reduction while preserving native protein structure and subsequent accessibility [60]. Similarly, in plant-based treatments, soybean protein isolates showed the highest emulsification when subjected to 85–90 °C for 2–4 min, conditions that partially reduced the exposure of glycinin molecules to the aqueous environment while avoiding excessive aggregation and reducing intermediate activity [61].

#### 5.6.2. Prevention of Over-Processing and Quality Degradation

Understanding PPI mechanisms prevents common overprocessing scenarios that compromise nutritional value and performance. In meat processing, excessive mechanical disturbance during comminution can lead to undesirable protein–protein interaction cross-linking reactions of cross-linked proteins, leading to the hardening of the structure and reduction of the preservative properties [62]. This mechanistic approach is in stark contrast to traditional experimental approaches that typically detect performance limitations only after system failure.

#### 5.6.3. Texture Consistency and Yield Enhancement

Proper control of PPIs directly improves product stability and increases production yield. In surimi production, proper management of the critical myosin interactions by controlling the washing steps and the addition of cryoprotectant preserves the gel-forming ability during cold storage, and reduces the yield loss from protein degradation by 12–18% [63]. The plant-based meat composites achieved fibrous structures under controlled extrusion conditions and promoted the consistency of soybean and pea protein composites through shear-induced training.

#### 5.6.4. Process Design and Equipment Selection

PPI behavior quickly informs fundamental decisions about tools and processes. The selection of high-shear mixing materials relies heavily on knowledge of the shear stress of target protein interactions. Egg white protein, for example, forms fixed networks at moderate shear but is irreversible in conditions of high shear rates exceeding 10,000 s^−1^. Knowledge of such limitations guides the choice between rotor–stator homogenizers, high-pressure homogenizers, and ultrasonic services based on the product-specific requirements of PPI rather than the general quality of the product. In terms of heat treatment, understanding the rate at which proteins aggregate with temperature reveals the choice between batch and continuity: proteins that exhibit slow aggregation (e.g., ovalbumin) perform well in continuous HTST systems, whereas rapidly aggregated proteins (e.g., blood) require protein degradation.

Translating laboratory findings to industrial-scale implementation presents persistent challenges. Most mechanistic studies are conducted at gram-scale with purified proteins, while industrial processes involve complex matrices at kilogram scale with variable raw material compositions. PPI behavior under continuous-flow industrial conditions (e.g., UHT tubular heat exchangers, twin-screw extruders) has been characterized far less rigorously than under batch conditions. Furthermore, comparisons across studies are complicated by the absence of standardized processing protocols and characterization methods, making it difficult to determine whether reported differences reflect genuine mechanistic variation or methodological inconsistency.

## 6. PPIs in Major Food Categories and Their Quality Implications

This demonstrates that protein–protein interactions differ in each food group which is due to the particular features, setup and processes involved in each. The different features of proteins call for special strategies to learn about and control PPIs in food for better final results.

### 6.1. Dairy Systems

There has been a lot of research on dairy systems, mostly because of the detailed interactions among casein and whey proteins. The interactions formed by κ-casein and β-lactoglobulin have been analyzed in greater detail than ever before, and the latest research reveals ways these relationships change the texture, flavor and stability of several dairy products [52]. It has been shown through recent studies that the pH level strongly affects how these proteins bond, and the pH range of 6.0–6.5 favors the strongest interactions among them [1].

Researchers are investigating the formation of protein networks in yogurt, as they affect how yogurt feels and the level of water it can retain. The speed of acidification during fermentation affects how proteins join in aggregates, so it is favorable to use slower acidification so as to form better networks and achieve a better texture. Sequential heat-induced denaturation of whey proteins leads to a topological network required for improving along with the mechanical properties of yogurt gels. Selective strains of bacterial cultures were shown to regulate protein interactions by producing bacterial exopolysaccharides that interact with protein networks to increase texture characteristics and decrease syneresis.

The ripening of cheese involves complex proteolytic alterations that can provide modification of protein interactions over time. Cheese ripening involves the coordinated activity of several proteolytic systems that progressively alter protein interaction networks. Primary proteolysis is carried out by residual coagulant enzymes, primarily chymosin (EC 3.4.23.4) and bovine pepsin, which cleave the αs1-casein chain at the Phe23-Phe24 bond, producing αs1-I casein and reducing gel stiffness as the casein network becomes more extensible. Secondary proteolysis is mediated by endogenous milk protease plasmin (EC 3.4.21.7), which preferentially hydrolyzes β-casein at the Lys28-Lys29 and Lys105-His106 bonds, releasing γ-caseins and proteose peptones that contribute to texture softening and flavor development. Starter and non-starter lactic acid bacteria (LAB) contribute additional peptidase activity through cell envelope proteinases (CEPs) and intracellular aminopeptidases, which further degrade intermediate peptides to free amino acids and flavor precursors. The balance among these enzyme systems determines the rate of network dissolution, the accumulation of bioactive peptides with potential ACE-inhibitory or antimicrobial activity, and the characteristic rheological evolution from a firm, elastic gel toward the soft, spreadable texture associated with mature cheeses such as camembert or brie [64] The role of calcium phosphate nanoclusters in mediating protein–protein interactions has also been well characterized and serve critical functions in maintaining cheese structure and regulating moisture migration. Steady advancements in membrane filtration and microfiltration have attracted a considerable amount of research working towards modifying protein composition and their interactions which will impact product outcomes. Membrane processing can selectively fractionate proteins, which affects the formation of protein complexes and consistently improves functional properties in dairy products. The specific applications and quantitative outcome in PPI modification is given in Table 3.

### 6.2. Meat and Muscle Foods

The textural properties and water-holding capacity of muscle foods are founded on the interactions that occur between myofibrillar proteins. Recent works have comprehensively represented how differing processing conditions affect the extraction and gelation of myofibrillar proteins significantly influencing the quality and yield [69]. The details of PPI modification from fresh to processed meat are described in Table 4.

Protein oxidation in processing and storage is a crucial form of PPI occurring in meat systems. Some researchers extensively documented the influence of oxidative modifications on the structure and interactions of proteins, which frequently results in undesirable changes in texture and water-holding capacity [74]. New antioxidant systems and packaging materials have been developed to prevent these oxidative changes [45].

Enzymes that are endogenous to meat are playing a more recognized role in the modulation of PPIs during aging and storage of meat. Calpain and cathepsin activities produce characteristic cleavage patterns, which result in altered networks of protein interactions, participating in tenderization processes [29]. The interaction between muscle proteins and non-meat ingredients has been studied in great detail, especially regarding the impact of starches, fibers, and hydrocolloids on protein network formation [75].

### 6.3. Seafood Proteins

Unlike terrestrial muscle proteins, marine proteins have different interaction properties. Fish myofibrillar proteins have distinct thermal stability and aggregation requirements which necessitate modified processing. Surimi products are one of the most commercially lucrative manifestations of protein–protein interactions in the aquaculture sector. The gel-forming capability of surimi depends very much on the thermally induced behavior of myofibrillar proteins—mainly myosin and actin. The gelation process follows a well-defined two-step mechanism: in the first step, heating at 40 °C for 30–60 min promotes the formation of a nascent protein network through cross-linking, hydrophobic interactions and disulfide bond formation between myofibrillar proteins; in the second step, elevation to 90 °C further promotes myofibrillar protein aggregation, resulting in a dense and elastic gel matrix, which is responsible for the increase in chewiness and water retention of the end product [76]. Crucially, the molecular forces at work in establishing the gel network’s stability, i.e., ionic bonds, hydrogen bonds, hydrophobic interactions, and the formation of disulfide bonds, are subject to a very pronounced sensitivity to the processing conditions: any perturbation in these interactions makes the gelatinous matrix as a whole less robust [4]. The thermal stability of fish myofibrillar proteins exhibits strong species dependence, presenting both challenges and opportunities for processing optimization. For cold-water species, such as Atlantic cod (*Gadus morhua*), this can occur as low as 44 °C, while for Atlantic salmon (*Salmo salar*), the values for myosin denaturation range from 45 °C to 65 °C, depending on the type of myofibrillar fibers. Tuna, on the other hand, exhibit a markedly higher myosin thermal stability than cold-water species, an adaptation reflecting their elevated body temperatures and warm-water habitat [77]. The differential denaturation temperatures observed among taxa directly determine the optimal processing window for every raw material. While a uniform heat treatment protocol provides better gelation in Alaska pollock surimi, a major raw material in the global surimi industry, application to warm-water fish could lead to higher aggregation and structural aberrations.

Moreover, different red and white myosin fibers may exhibit different characteristics of denaturation within a single species. In this case, the red myosin of Atlantic salmon has a higher transition temperature than the white one, as determined by rheological experiments, thus illustrating isoform specific structural differences and distinct susceptibility to heat-induced unfolding and intermolecular cross-linking [77]. These species and muscle type dependent differences show the need to calibrate processing factors with respect to the molecular characterization of the raw materials, and not to employ a general heat treatment schedule.

In addition to the thermal processing, the post-mortem biochemical evolution represents a crucial aspect in the management of the quality of proteins in seafood matrices. Upon cessation of respiration when fish are dead, aerobic pathways are stopped and anaerobic glycolysis takes place, resulting in lactic acid production, and the resulting decrease in pH [78]. A reduction in pH has a pronounced effect on protein–protein interactions. As the pH approaches the isoelectric point of myofibrillar proteins, the electrostatic repulsion among the protein molecules is diminished and it leads to protein aggregation, partial denaturation of proteins, and a significant reduction of water-holding capacity. Contemporary proteomic explorations proved that label-free quantitative proteomics can identify certain degradation products of myofibrillar proteins and release them as molecular indicators of fish freshness, thus providing a mechanistic basis for the establishment of process-adaptive quality-control processes in industrial seafood processing.

### 6.4. Plant-Based Proteins

As the market for plant-based products continues growing, research on plant protein functionality has increased. The aggregation and gelation properties of prominent plant proteins such as pea, soy, and wheat have been extensively described, with important distinctions to animal proteins that affect processing needs and final product characteristics as described in Table 5 [79]. Also, in order to obtain similar gel strength as animal proteins, plant proteins need higher temperatures as well as specific pH conditions [79].

### 6.5. Alternative Protein Sources (Microalgae, Fungi, Insects)

The interaction and food system potential of alternative protein sources such as microalgae, fungi, and insects has been widely researched. Characterization studies observed the aggregation tendency of microalgae proteins during processing, which is likely caused by a high rate of polar amino acids in the microalgae proteins and thus would require specific processing conditions. Also, due to their amino acid profiles and amphiphilic nature, insect proteins have good emulsifying properties. Fungal proteins, especially those of the species related to Fusarium, also show great potential in generating fibrous structures based on their control over protein interactions.

Characterization of the functionality of plant proteins in the presence of anti-nutritional factors have resulted in better processing. Some particular enzymatic treatments could lower tannin and phytate levels, while maintaining their ability to interact with proteins. The creation of hybrid protein systems, which consist of combinations of plant proteins from different sources or blends of plant and animal proteins, has been presented as a viable strategy to obtain better functionality and nutritional profiles.

The protein extraction methodology has a key influence on the interaction characteristics of plant proteins. Indeed, alkaline solubilization and isoelectric precipitation could modify protein structure and behavior in terms of interactions. Enzymatic or mechanical extraction methods offer potential alternatives to this processing which are believed to better maintain native protein structure and functionality [27].

### 6.6. Fermented Foods

Fermentation technologies for foods are distinctive in that microbial enzymes have a profound effect on protein interactions over time. Studies conducted on products of traditional fermentation have uncovered intricate interactions that arise throughout the fermentation process. Investigations through fermented soy products showed how certain proteolytic enzyme activities can generate peptides with more functional properties as well as behaviors towards various interactions [85]. The involvement of microbial transglutaminase in fermented food products has been described, and it is known to play a role in texture development, especially in tempeh and natto.

Other fermented dairy products, like kefir and buttermilk, have gained interest. The particular microbial consortium was found to produce specific aggregation behavior for the proteins in kefir, resulting in unique textural characteristics. Fermentation studies conducted with plant-based alternatives have demonstrated the potential of controlled fermentation to enhance protein functionality and lower anti-nutritional factors [86].

Manipulating protein interactions in the engineering of structured foods is becoming a relevant field of research. Research exemplified the potential of controlled interactions between proteins to form intricate and hierarchical structures in meat analogs. Through high-moisture extrusion and protein interactions specifically modulated, products have been developed that can achieve fibrous textures similar to traditional meat [4].

Promising advances have been made in edible packaging applications based on interactions with proteins. In this regard, scientists proved the ability of certain protein networks to develop films with barrier properties for food packaging and that were completely biodegradable. The interaction of various proteins in composite films improves their mechanical properties and their range of applications [85].

This compilation of different studies from various foods illustrates how protein–protein interactions are critical in all categories of food and signifies the particular needs and possibilities that exist in the different types of food systems. Such knowledge has provided a basis for food products development, and, improving quality in all other principal food categories.

A recurring limitation across all food categories is the reliance on single-protein model systems. Most mechanistic insights derive from studies of isolated whey protein, gluten, or soy protein isolate, whereas real food matrices contain multiple proteins interacting simultaneously with lipids, polysaccharides, minerals, and phenolic compounds. These co-solutes can profoundly alter PPI pathways, sometimes synergistically and sometimes antagonistically, yet multi-component interaction studies remain underrepresented in the literature. This gap represents a critical bottleneck in translating fundamental PPI knowledge into predictive processing models.

## 7. Applications of PPIs in Improving Food Quality

Applying knowledge of protein–protein interactions has enabled improved control under specific conditions over different aspects of food quality, affecting how food scientists create and enhance products. The strategic modulation of PPIs has demonstrated utility in preserving color stability, enhancing flavor delivery, engineering texture, improving nutrient bioavailability, and ensuring food safety.

### 7.1. Color Stability

Visual appeal is a primary determinant of consumer acceptability, and studies have found that PPIs can be controlled to improve their colors. Particular protein–protein interactions make natural pigments more stable due to the protective covering [86].

Meat products get their proper color and stability after myoglobin and other proteins interact. The researchers found that controlling proteins in foods can help form stronger oxymyoglobin groups which helps fresh meat maintain its attractive red color. It has been shown that when specific protein cross-linkers are used, protective networks develop around color-sensitive parts in processed meats, giving them considerably more color stability [87].

Constructing and sustaining the desirable colors of plant-based meat is a major difficulty. Research highlights that engineering protein links can stabilize plant-based heme analogs during thermal processing, preserving their characteristic meat-like coloration. Using protein–phenolic complexes allows plant proteins to develop quicker and more natural browning enabling plant-based burger products to exhibit browning characteristics resembling conventional meat upon cooking [87].

### 7.2. Flavor Delivery

Linking the shape of a protein to how it delivers flavor is one of the most complex examples of how protein–protein interactions (PPIs) work. Subtle changes in protein shape can create binding sites for flavor compounds, enabling controlled flavor release during oral processing [88]. Research revealed that making protein flavor conjugates via controlled Maillard reactions helps to form unique flavors and make flavor more stable [28].

Encapsulating volatile flavor molecules within protein matrices has become an important technique for maintaining flavor stability and releasing them over time. Protein engineering technology can create microspheres that allow flavor compounds to be gradually released as they move. It has been established how amino acids within proteins interact with taste compounds to improve the umami taste and reduce bitterness in plant ingredients [89].

Bitterness has been removed from plant proteins by changing the parts where bitter compounds bind. Study revealed that specific protein–protein interactions can alter structures that lower binding to bitter compounds by approximately 70% [88]. Enhancements in alternative protein products’ flavor have been made by applying controlled enzymatic changes to proteins [89].

### 7.3. Texture Engineering

Modifications to texture today use PPI more expertly, producing textures that more closely mimic those found in animal products. Multi-stage protein interactions allow for the creation of products that can range from mildly cohesive to fully fibrous, according to different length scales. Emerging gradient protein networks are behind the development of foods that have different textures as they are eaten [74].

Proteins are now finely managed to achieve preferred textures in different kinds of foods. Changes in the arrangement of proteins due to controlled cross-linking have resulted in products that behave differently when stressed in different ways [32]. Such proteins are manipulated based on temperature to gain textures that can shift and change over time. Experiments revealed that giving proteins a specific form, changes the product from firm to creamy when exposed to mouth temperature. Through combining proteins with phase-change materials, scientists have been able to develop products that change their texture as their temperature changes [90].

### 7.4. Nutrient Bioavailability

Improving the way proteins interact can boost the availability of nutrients in food. Certain protein–mineral complexes have been shown to enhance mineral bioavailability by up to threefold compared to minerals on their own. It has been demonstrated that forming complexes between vitamins and proteins helps to secure sensitive vitamins and raises their bioavailability in the food [91]. The process of controlled hydrolysis has become better, allowing for the creation of bioactive peptides with health-promoting properties. Studies have demonstrated that enzymatically modified peptides can exert beneficial effects on blood pressure, oxidative stress, and inflammation while preserving functional food properties. Today, thanks to encapsulating probiotics in protein matrices, there are probiotic products on the market that survive for longer and are sent to selected parts of the digestive tract.

Special systems using protein are being developed as a way to resolve the problem of bioavailability for nutraceuticals. Results from experiment proved that using proteins can create capsules that protect drugs in the stomach and deliver them safely into the intestines [92].

### 7.5. Food Safety and Antimicrobial Systems

Antimicrobial protein systems have led to a much greater level of food safety. Recent investigations have demonstrated that targeted modification of antimicrobial proteins can inhibit bacterial activity and preserve food quality [78]. These protein-based antimicrobial films have shown to be quite effective at fighting foodborne pathogens, reaching a level of 99.9% pathogen reduction [93].

### 7.6. Allergen Reduction and Health Applications

Using protein modification to minimize allergens is now a vital safety approach. Changes in proteins may both prevent allergic reactions and preserve their techno-functional properties. Scientists have altered the texture of many foods by studying PPIs and developing the right proteins and their interactions. To achieve authentic flavor profiles in dairy alternatives and meat analogs, scientists now regularly use different methods to control protein aggregation happening at different levels [89]. To achieve acceptable sensory properties in gluten-free formulations, food scientists make use of enzymes to also improve the consistency of yogurts and similar dairy products. PPI-guided formulations can produce products with ingredients that align with consumer preferences. The mixing of physical and chemical treatments with protein gelation has led to the discovery of unique textures in various food goods. Food manufacturers achieve this by arranging proteins in specific ways enabling products with superior textural and sensory properties.

PPI-based approaches to nutritional enhancement have advanced significantly. Extensive studies have been done on the effects of PPIs on digestion which has led to identifying formulations that substantially enhance protein digestibility and absorption in many kinds of food. Reviewing the impact of tannins and phytates on proteins has suggested methods to remove and prevent these factors while saving the helpful benefits of protein interactions [24]. Because bioactive peptides can be produced using accurate modifications to proteins, a variety of technological advances have been introduced in healthcare, agriculture and other fields. Many efforts are made to modify proteins by making them less allergic and still maintaining their quality and value for health. Relying on special protein-based materials allows for delivering bioactive elements correctly to different parts of the digestive system for better efficiency.

### 7.7. Stability Enhancement in Processing and Storage

Advanced use of PPI plays a major role in boosting food system stability. Researchers are focusing on designing methods to make protein-stabilized emulsions more heat-resistant with accurate control over how emulsions are built and how the proteins are arranged at the interface. Thorough research has made it simpler to prepare thermally processed beverages and infant formulas with balanced nutrition and while preserving desirable sensory attributes. Changing the way proteins interact with each other has managed to stop texture breakdown and phase separation during freeze–thaw processes in frozen desserts. Preventing large ice crystals is mainly done by creating stronger protein structures called cryoprotectants [6]. Well-planned modifications of protein surfaces on molecules have greatly boosted the stability and usage of protein-based foams in the food industry.

Strategic PPI modulation enhances the sensory properties of high-protein foods. Various studies have proven that manipulating protein networks helps keep essential nutrients and makes food taste and smell better [18]. Thorough studies have pointed out the important roles that proteins play in making foods creamier and smoother to the mouth. These discoveries have enabled dairy-free beverages and protein-rich products to achieve improved flavor profiles with significantly reduced off-notes. Modifications to the way proteins interact with flavors have made it possible for these products to taste better and have less off-flavor and unpleasant notes. Further knowledge about how proteins break down during eating has allowed for the creation of foods that satisfy customers’ taste and texture needs.

## 8. Emerging Trends and Future Perspectives

Rapid progress in the development of PPIs in food systems is being promoted by advances in new technologies and the evolving taste preferences of customers. Computer-driven predictions of protein interactions in food are leading to the development of novel food science. Advances in machine learning and molecular modeling have contributed to the understanding of the proteins responses across diverse foods systems [9]. These computational tools may be valuable in formulations of protein ingredients having select interaction characteristics. High-throughput experiments to validate predicted solutions are an effective approach to protein-based food development. Artificial intelligence has made it possible to reveal many long-hidden relationships between how a protein is constructed and how it works.

Researchers are working to engineer proteins with precisely defined structural and functional properties. It has been proved by experiments that it is possible for genetic engineering to succeed in improving the functional quality of food protein components. Whilst there are challenges for its acceptance, this approach continues to present new opportunities for controlling PPIs in food products. The rise of cell-free protein production means that proteins can be custom-designed with precise functional specifications. Utilization of synthetic biology to design food proteins may provide new prospects for overcoming protein source functional problems.

Researchers are also examining more closely the effects of proteins and other food components on the body. These advances have enabled a reduction in dietary protein requirements while improving food quality outcomes. Multi-component structured inclusions have enabled the production of food mixtures with enhanced stability and performance. It remains difficult to know how these interactions change when food is processed, stored, and ingested.

Scientists conducted research on energy-efficient approaches for managing PPIs recently. Cold-set gelation reduces energy consumption by enabling network formation at relatively mild temperatures. Food manufacturers are prioritizing processing strategies that preserve protein functionality and safety. One of the features of this technology is the use of processing methods that do not excessively interfere with proteins and ensure safety during the processing. Efforts to save water and energy and maintain the desired PPI are as vital today as they were before. Despite significant progress, controlling protein–protein interactions (PPIs) in complex foods remains challenging.

## 9. Conclusions

This review focuses on protein–protein interactions (PPIs) as a basis for determining structure–function–quality relationships in food systems. By combining the synthesis of molecular mechanisms, mesoscale assembly, process control and industrial results, we propose a multi-scale framework for the recognition of the importance of mechanistic insights into PPIs for the predictive and rational design of food products. Accordingly, food engineering strategies in the future need to go beyond experimental-based strategies of optimization, but need to include models at cross scales that are anchored to molecular interaction dynamics and measurable quality attributes.

### 9.1. Summary and Current Limitations

This review consolidates mechanistic and applied knowledge of protein–protein interactions across dairy, meat, seafood, plant-based, and fermented food systems. Analytical advances, particularly in high-resolution mass spectrometry, cryo-electron microscopy, and interfacial rheology, have substantially improved the resolution at which PPIs can be characterized in complex matrices. Processing technologies ranging from conventional thermal treatment to high-pressure processing, ultrasound, and pulsed electric fields have each been shown to produce distinct PPI outcomes with direct implications for texture, stability, flavor delivery, and nutritional bioavailability. Nevertheless, several limitations constrain the field. Most mechanistic data derive from single-protein model systems at laboratory scale, limiting direct translation to industrial multi-component matrices. The absence of standardized characterization protocols across studies makes cross-study comparisons unreliable. Predictive models linking mesoscale PPI structure to macroscopic sensory attributes remain insufficiently validated for industrial use.

### 9.2. Future Research Priorities

Future research should prioritize three directions. First, the development of multi-component PPI models that account for the simultaneous presence of proteins, polysaccharides, lipids, and phenolics under processing-relevant concentrations. Second, the expansion of mechanistic studies to alternative protein sources, including microalgae, insect-derived proteins, and fungal proteins, which exhibit interaction behavior substantially different from conventional animal and plant proteins. Third, the integration of machine learning approaches with high-throughput analytical platforms to accelerate the translation of molecular-level PPI data into actionable processing guidelines. Progress along these lines will provide food scientists with the quantitative tools required to engineer protein networks with defined functional properties across a broad range of food applications.

Alternative protein sources, including microalgae, insect-derived proteins, and proteins from precision fermentation, present distinct PPI challenges that have not yet been addressed with the depth applied to conventional dairy and meat systems. Microalgae proteins, rich in polar amino acids, show pronounced aggregation sensitivity to pH and ionic strength changes during downstream processing that current analytical frameworks cannot fully resolve. Insect proteins, such as those from Acheta domesticus and Tenebrio molitor, exhibit strong amphiphilic character and cross-react structurally with crustacean allergens, raising both functional and safety questions requiring mechanistic clarification. Cultured meat protein networks, assembled in vitro from myosatellite cell-derived myofibrillar proteins, must achieve the hierarchical anisotropic structure of native muscle tissue through processing-induced PPI engineering, a challenge fundamentally different from conventional meat restructuring. The analytical methods reviewed in this paper, particularly cryo-TEM, XL-MS, and interfacial rheology, are well positioned to address these questions, and their systematic application to alternative protein matrices should be a priority for the next research cycle.

## Figures and Tables

**Table 1:** Analytical method comparison for PPI characterization in food systems.

Method	Information Provided	Best Application	Limitations	Recent Advancement (2020–2026)	References
FTIR spectroscopy	Secondary structure (α-helix, β-sheet, random coil), amide I & II bands, aggregation state	Thermal processing monitoring, protein conformation changes, real-time denaturation kinetics	Limited spatial resolution, bulk measurement, requires spectral deconvolution, sensitive to water interference	ATR-FTIR imaging with 10 μm spatial resolution, enables protein distribution mapping in heterogeneous gels	[6]
Circular dichroism (CD)	Protein secondary structure quantification, thermal stability curves, folding/unfolding transitions	Conformational analysis in solution, pH/temperature stability screening, protein–protein complex formation	Dilute samples required (0.1–1 mg/mL), turbidity interference, path length limited to 0.1–1 cm	Synchrotron radiation CD (SRCD) with 100× higher photon flux, enables low-concentration measurements and complex matrices	[7]
Confocal laser scanning microscopy (CLSM)	Protein network visualization, pore size distribution, 3D structural architecture, spatial protein distribution	Gel microstructure analysis, emulsion droplet characterization, foam lamella imaging, protein network topology	Fluorescent labeling required, potential artifacts from dyes, depth penetration <200 μm, sample preparation critical	Super-resolution CLSM (STED, SIM) achieving 10–50 nm resolution, reveals nanoscale protein aggregates previously invisible	[8]
Atomic force microscopy (AFM)	Surface topography, nanoscale structure, protein aggregate morphology, mechanical properties (nN force)	Protein fibril characterization, surface roughness, nanoparticle imaging, protein–protein interaction force measurement	Surface-limited, slow scanning (30 min/image), requires flat samples, tip artifacts possible, expensive equipment	High-speed AFM (HS-AFM) enables dynamic imaging of protein assembly in real-time (10–100 frames/s)	[9]
Cross-linking mass spectrometry (XL-MS)	Protein–protein interaction interfaces, cross-link sites identification, spatial proximity constraints (<30 Å)	Protein complex topology mapping, interface residue identification, conformational ensemble characterization	Complex sample preparation, requires MS/MS expertise, false positives possible, limited to Lys-Lys or Cys-Cys pairs	Cleavable cross-linkers (MS-cleavable, CID-cleavable) simplify spectra interpretation, improve identification confidence	[10]
Hydrogen–deuterium exchange MS (HDX-MS)	Protein dynamics, solvent accessibility, conformational changes upon binding, protein–protein interface mapping	Folding/unfolding studies, binding site identification, stability comparison, conformational dynamics in PPI	Requires deuterated systems, time-resolution limited (seconds), back-exchange during analysis, expensive instrumentation	Millisecond HDX-MS with microfluidic mixing achieves <10 ms time resolution, captures transient intermediates	[11]
Native mass spectrometry (Native MS)	Intact protein complex mass, stoichiometry, binding affinity (Kd), oligomeric state distribution	Protein complex characterization, oligomerization assessment, ligand binding studies, weak interaction analysis	Volatile buffer required, limited to <800 kDa complexes, ionization bias possible, gentle ionization needed	Charge detection MS (CD-MS) extends mass range to MDa scale, enables characterization of large protein assemblies	[12]
Molecular dynamics (MD) simulation	Atomic-level interaction dynamics, binding free energy, conformational sampling, interaction kinetics	Mechanism prediction, mutation effect forecasting, solvent effect analysis, temperature/pH simulation	Computationally expensive, force field accuracy dependent, timescale limited (typically <1 μs), validation required	AI-enhanced MD (AlphaFold-Multimer integrated) predicts protein complex structures, reduces simulation time 100-fold	[13]

**Table 2:** Processing technology impact matrix: PPI modifications and industrial outcomes.

Technology	Food System	PPI Modification	Quality Improvement	Shelf-Life Impact	Industrial Adoption Status	References
High pressure processing (HPP)	Fresh juice with added whey protein (5% *w*/*v*)	Maintains native structure at 400 MPa, limited unfolding, prevents aggregation, preserves solubility 95%	Color retention +40% (ΔE <3 vs. 8), vitamin C preserved 92% vs. 65%, protein functionality maintained	Microbial reduction 5-log, shelf life +60 days refrigerated (90 vs. 30 days)	15% premium juice market (USA, EU), growing 12%/year, major adoption by Suja, Evolution Fresh	[34]
Ultrasound treatment	Surimi gel (Alaska pollock)	Cavitation-induced protein unfolding, enhanced disulfide exchange, myosin cross-linking +42%	Gel strength (breaking force) +35% (18 N → 24 N), WHC +28%, whiteness L* 82 vs. 78	+14 days refrigerated (21 vs. 7 days), reduced drip loss 12% → 4%	30% in Asian surimi market (Japan, Thailand), standard in premium products, proven 10-year track record	[35]
Microwave heating	Restructured ham (70% pork, 30% water)	Rapid heating (2.45 GHz), volumetric protein denaturation, uniform myosin gelation, reduced aggregation	Uniform texture, reduced cooking loss 8% vs. 15%, improved slicing, moisture distribution ±2% vs. ±8%	No significant change (same 45 days), but reduced processing time 80% (12 min vs. 60 min)	40% in industrial meat processing (USA, EU), rapid adoption due to energy efficiency, proven for 15+ years	[36]
Infrared heating	Tofu production (soybean curd)	Surface protein gelation, controlled heat penetration, gradient network formation, minimal over-processing	Smooth surface texture, firm gel (hardness 4.2 N), reduced brittleness, improved yield +8%	+7 days refrigerated (14 vs. 7 days), reduced surface bacterial growth	20% in Asian tofu production (China, Japan), growing for premium products, energy-efficient alternative	[37]
Pulsed electric field (PEF)	Liquid egg white (11% protein)	Electroporation, limited unfolding at 20–40 kV/cm, maintains β-sheet structure, prevents aggregation	Preserved foaming capacity 88% vs. 45% (heat), angel food cake volume +12%, maintained clarity	Microbial reduction 4-log, pasteurized quality, +21 days refrigerated (35 vs. 14 days)	5% specialty egg market (EU, USA), limited but growing, regulatory approval in EU 2020, USA GRAS status	[36]
Precision fermentation (engineered microbes)	Animal-free whey protein (identical to bovine)	Produces native β-lactoglobulin, identical PPI behavior, natural disulfide bonding, heat-induced gelation	Identical functionality to dairy whey, superior allergen control, customizable post-translational modifications	Same as conventional whey (6–12 months dry), superior microbial quality, no farm contamination risk	Emerging market, Perfect Day (USA) 2020 launch, regulatory approval USA/Singapore, scaling to commodity volumes	[38]

CAPEX = capital expenditure; OPEX = operating expenditure; ΔE = color difference (ΔE <3 imperceptible to human eye).

**Table 3:** PPI mechanisms in dairy products: specific applications and quantitative outcomes.

PPI Mechanism	Product	Processing Condition	Quantitative Outcome	Quality Impact	Reference
Hydrophobic interaction + limited disulfide bonding	Greek yogurt (strained)	Heat: 85 °C/30 min, pH 4.6, culture addition at 42 °C	WHC increased 34%, syneresis reduced from 15% to 4.2%, protein network density +28%	Firmer texture (hardness 0.8 N → 1.9 N), reduced whey separation, improved mouthfeel	[48]
Calcium-mediated cross-linking + disulfide bonds	Mozzarella cheese	Stretching: 57 °C, pH 5.2, calcium chloride 0.02%, mechanical working 5 min	Protein network strength +45%, stretch ability 180%, Ca2+ binding sites 12 per casein molecule	Improved melt (flow distance 8.5 cm at 280 °C), enhanced stretchability, characteristic fibrous structure	[65]
Electrostatic repulsion maintenance (limited aggregation)	UHT milk	Heat: 140 °C/4 s, pH 6.7, homogenization 200 bar, κ-casein stabilization	Large aggregate formation reduced 62%, particle size <200 nm maintained, age gelation delayed	Extended shelf life (6 → 9 months), minimal sediment, maintained color (L* value 82 vs. 79)	[66]
Cold gelation via electrostatic screening	Acid-induced whey protein gel (ricotta-type)	Pre-heat 85 °C/30 min, cool to 20 °C, acidify to pH 4.8 with GDL, ionic strength 50 mM	Gel strength (G′) 180 Pa, WHC 92%, pore size 2–8 μm, network formed at 25 °C	Fine texture, high moisture retention, low syneresis (<3%), smooth mouthfeel	[67]
Enzymatic cross-linking (transglutaminase)	Set-type yogurt (fortified protein)	TGase 0.5 U/g protein, 40 °C/2 h, then culture fermentation pH 4.5	Gel firmness +68%, viscosity 4200 → 8900 cP, ε-(γ-glutamyl) lysine bonds 18 per 100 residues	Reduced syneresis (8% → 2%), enhanced creaminess, improved protein content delivery (12 g/100 g)	[68]

WHC = water-holding capacity; GDL = glucono-delta-lactone; TGase = transglutaminase; G′ = storage modulus.

**Table 4:** Meat product PPI modifications: from fresh to processed.

Product	PPI Modification	Processing Conditions	Protein Network Result	Texture & Quality	Reference
Fresh beef steak (ribeye)	Aging-induced proteolysis, calpain degradation of Z-disks, limited myofibrillar breakdown	Wet aging 14 days at 2 °C, vacuum-packed, pH 5.6, then grilling 200 °C to 60 °C core	Weakened myofibrillar structure, troponin-T degradation 65%, desmin breakdown 40%, Z-disk disruption	Tenderness improved (WBSF 28 N → 18 N), juiciness score 7.8/10, flavor development, moisture loss 22%	[64]
Emulsified sausage (frankfurter)	Salt-soluble protein extraction, myosin–actin gelation, fat emulsification by protein coating	Chopping with 2% NaCl, temperature <12 °C, fat 25%, stuffing, cooking to 72 °C core	Continuous protein gel matrix encapsulating fat, myosin denaturation 85%, protein–fat interface 15 m^2^/g	Firm snap texture (8 N bite), juicy (fat retention 98%), smooth mouthfeel, yield 98%, shelf life 60 days	[70]
Dry-cured ham (prosciutto)	Salt-induced protein dehydration, proteolysis by cathepsins, slow myofibrillar degradation	Salting 3–4 weeks (3% NaCl), washing, drying 12–18 months at 14–18 °C, 70–75% RH, final aw 0.85	Concentrated protein matrix, moisture reduced 65% → 40%, extensive proteolysis (free amino acids 8–12 g/kg)	Firm slicing texture (hardness 45 N), intense flavor, characteristic aroma, shelf-stable, no cooking required	[71]
Restructured chicken breast (formed)	Transglutaminase cross-linking of myofibrillar proteins, cold-set binding, covalent isopeptide bonds	Chicken pieces, TGase 0.5%, mixing 10 min at 4 °C, forming under pressure, refrigeration 12 h, cook 75 °C	Protein–protein cross-links bind pieces, ε-(γ-glutamyl) lysine bonds 15 per 100 k Da, continuous structure	Whole-muscle appearance, sliceable, hardness 25 N, improved yield +8%, reduced cook loss 18% vs. 28%	[72]
Sous-vide beef tenderloin	Controlled low-temperature myosin denaturation, minimal actin denaturation, collagen partial gelatinization	Vacuum-sealed, 56 °C water bath 2 h, then searing 250 °C surface 1 min/side, core remains 56 °C	Intact actin filaments, myosin coagulated, minimal protein aggregation, collagen softened 30%	Extremely tender (WBSF 12 N), juicy (moisture retention 88%), uniform doneness, pink color throughout	[73]

WBSF = Warner–Bratzler shear force; aw = water activity; RH = relative humidity; TGase = transglutaminase.

**Table 5:** PPI in plant-based meat alternatives: processing–structure–quality relationships (plant-based proteins).

Protein Source	PPI Modification	Technology & Conditions	Structural Change	Texture Outcome	References
Pea protein isolate (85% protein)	Transglutaminase cross-linking	Enzymatic: 0.8 U/g, 50 °C, 6 h, pH 7.0, then high-moisture extrusion 140 °C	Fibrous network formation, protein alignment index 0.68, cross-link density 22/100 kDa	Meat-like chewiness, hardness 2.1 N, springiness 0.81, fibrous tear pattern	[80]
Soy protein concentrate (70% protein)	HPP-induced controlled aggregation	High pressure: 600 MPa, 5 min, 25 °C, pH 7.2, then heating 75 °C/10 min	Dense protein matrix, aggregate size 0.8–3.5 μm, β-sheet content +18%	Juiciness +28%, springiness 0.82, cohesiveness 0.71, beef-like texture	[81]
Wheat gluten + pea protein blend (60:40)	Shear-induced alignment + thermal gelation	High-moisture extrusion: 130 °C, screw speed 250 rpm, moisture 55%, cooling die 40 °C	Anisotropic fibrous structure, fiber length 15–45 mm, alignment ratio 3.8:1	Directional bite, hardness parallel: 3.2 N, perpendicular: 5.8 N, chicken-like	[82]
Faba bean protein isolate (90% protein)	pH-shifting-induced gelation	Alkaline extraction pH 11, protein precipitation pH 4.5, neutralization, heat 85 °C/20 min	Fine-stranded gel network, pore size 1.5–6 μm, storage modulus 850 Pa	Soft gel texture, WHC 89%, low hardness 0.9 N, suitable for emulsion products	[83]
Mycoprotein (Fusarium venenatum)	Native hyphal alignment + heating	Fermentation-grown hyphae, steam texturization 95 °C/15 min, hyphal preservation	Natural fibrous structure, hyphal diameter 3–5 μm, length 100–500 μm, native alignment	Meat-like bite, hardness 2.8 N, high juiciness, fibrous tear, minimal processing	[84]

HPP = high-pressure processing; n = number of panelists. Consumer scores based on 10-point hedonic scale.

## Data Availability

No new data were created or analyzed in this study. Data sharing is not applicable to this article.

## Abbreviations

FTIR	Fourier-Transform Infrared Spectroscopy
HPP	High-Pressure Processing
PPI	Protein–Protein Interaction
CGC	Critical Gel Concentration
SAXS	Small-angle X-ray Scattering
DF	Fractal Dimension
pI	Isoelectric Point
WHC	Water-Holding Capacity
DLS	Dynamic Light Scattering
DSC	Differential Scanning Calorimetry
CD	Circular Dichroism Spectroscopy
XL-MS	Cross-linking Mass Spectrometry
HDX-MS	Hydrogen–Deuterium Exchange Mass Spectrometry
